# Mechanical Behavior and Damage Characteristics of Cemented Tailings Backfill Under Multiple Different Stress Disturbances

**DOI:** 10.3390/ma19122654

**Published:** 2026-06-20

**Authors:** Xiaofei Li, Yuanfan Liu, Jie Wang, Yan Li, Jianxin Fu

**Affiliations:** 1 School of Resources and Safety Engineering, University of Science and Technology Beijing, Beijing 100083, China; lixiaofei@sd-gold.com (X.L.); fujun0011@126.com (J.F.); 2 Shandong Gold Group Co., Ltd., Jinan 250100, China

**Keywords:** composite disturbance, cemented tailings backfill, energy evolution, micro-damage, heterogeneous mechanisms

## Abstract

To investigate the impact of underground multiple stress disturbances on the long-term stability of cemented tailings backfill (CTB), this study conducted experiments under different disturbance levels (20–80% of static strength) and frequencies (1–4 times). By comprehensively utilizing mechanical testing, wave velocity monitoring, digital image correlation (DIC), and scanning electron microscopy (SEM), the “heterogeneous” evolution mechanism of macro-micro damage was revealed. The results indicate that disturbance level and frequency exert distinctly different driving effects on the deterioration of CTB, rather than a simple linear superposition. Specifically, low-frequency disturbance produces a compaction strengthening effect, microscopically promoting the generation of Ca(OH)_2_ and ettringite (increased Ca/Si ratio). In contrast, the combination of high disturbance and high frequency induces free water extrusion and inhibits hydration, leading to an advanced damage threshold based on energy evolution and the accelerated coalescence of microcracks, which favors the formation of C-S-H gel (decreased Ca/Si ratio). Within this heterogeneous mechanism, the disturbance level acts as the dominant controlling factor. This study clarifies the nonlinear mechanical and chemical evolution paths under composite disturbances, providing theoretical support for the dynamic stability control of backfill in deep multi-step mining.

## 1. Introduction

As deep, high-stress mining becomes the norm, the mechanical environment in which backfill bodies are located is becoming increasingly complex and harsh [1,2]. As the core structure of the backfilling mining method, cemented tailings backfill (CTB) plays an irreplaceable role in controlling ground pressure, ensuring the stability of the goaf, and achieving environmentally sound disposal of tailings. During critical processes such as two-step mining, frequent blasting and excavation activities inevitably subject the backfill to multiple, multi-level dynamic load disturbances from the surrounding rock mass [3,4]. These disturbances are not isolated single events but repeatedly act on the backfill at specific levels (disturbance intensity) and frequencies. They not only directly induce the evolution of macroscopic mechanical properties but also affect long-term stability by altering internal hydration processes, microcrack networks, and energy dissipation pathways [5,6]. However, existing studies have largely focused on single high-intensity impacts or fatigue loading with constant amplitude, with insufficient attention paid to complex multiple stress disturbances loading paths that more closely resemble the “multiple, variable-amplitude” stress disturbance sequences encountered in actual engineering applications [7,8]. Therefore, systematically elucidating the macro-micro response patterns and damage evolution mechanisms of CTB under various combinations of disturbance levels and frequencies is a critical scientific issue that urgently needs to be addressed to accurately predict mining stability, optimize backfill design parameters, and achieve safe and efficient mining operations [9,10].

Researchers both at home and abroad have conducted relevant studies on the mechanical behavior of fillers under dynamic loads. Chung et al. [11] established a dynamic model of a gear transmission system filled with damping particles within the gear bore by coupling multi-body dynamics (MBD) with the discrete element method (DEM), revealing that contact friction is the dominant mechanism of energy dissipation under centrifugal conditions. Duarte et al. [12] adopted numerical simulation methods to investigate the mechanical behavior of short rubber-modified concrete-filled steel tubes under combined cyclic bending and monotonic compression, evaluating the influence of rubber concrete on the energy dissipation capacity of the members. Mansour et al. [13] conducted three-dimensional nonlinear finite element analysis to investigate the numerical response of concrete-filled steel tube (CFST) columns reinforced with externally wrapped FRP composites under cyclic loading, analyzing the enhancement effects of FRP thickness and arrangement on the member’s lateral load-carrying capacity and energy absorption performance. Xue et al. [14] pointed out that the synergistic preparation of filling materials with lithium slag and tailings can alleviate damage propagation under impact loads. Comparative analysis showed that the proposed modified damage constitutive model reduces prediction errors by more than 50% compared with the unmodified model. Yang et al. [15] found that with the increase in impact load, the dynamic peak stress of filling materials increases linearly, the strain decreases linearly, and the secant modulus increases exponentially. Xie et al. [16,17] carried out Hopkinson bar tests and found that peak stress exhibits a significant increase as coarse aggregate content grows, with the maximum strength of filling materials increasing by 53.56%; the increase of mass fraction and cement/tailing ratio (CT ratio) exerts a significant strengthening effect on the strain rate effect of compressive strength. Song et al. [18] revealed the mesoscopic structural response law of filling materials under dynamic loads. The degree of mesoscopic structural deterioration rises with the increase of impact amplitude, and the compressive strength and elastic modulus present a three-stage variation trend of slow decrease, rapid decrease, and slow decrease successively.

Qiu et al. [19] investigated the mechanical properties of grouted fill under cyclic loading and found that the strength of the grouted fill under equal-amplitude cyclic load-unload behavior was generally higher than that under constant-amplitude cyclic load-unload behavior; it increased along with rising amounts of cycles, and the degree of damage to the grouted fill continuously deteriorated. The damage evolution for consolidated backfill under repeated loading exhibits three distinct stages, with acoustic emission signals displaying a “peak-trough” phenomenon, and cumulative ringing counts and cumulative energy showing a stepwise upward trend [20]. Wang et al. [21] found that when the combined bearing capacity of surrounding rock and backfill exceeds the historical maximum value during loading, new cracks initiate, leading to a sharp drop in the average infrared radiation temperature (AIRT); during unloading, the cracks suffer tensile deformation and induce another sharp decrease in AIRT. Affected by elastic energy release and frictional heat effects, the AIRT generally presents an overall rising trend throughout the loading and unloading process. Chen et al. [22]. conducted uniaxial cyclic loading-unloading tests on sandstone combined with wave velocity and acoustic emission monitoring, identified three precursors of rock failure, and found a linear correlation between wave velocity reduction and dissipated energy as well as anisotropic wave velocity variation. Festugato et al. [23] investigated the cyclic shear response of fibre-reinforced cemented paste backfill through cyclic simple shear tests and found that fibre addition could modify the shear stress–strain response and improve the ability of backfill to resist repeated shear deformation, while Suazo et al. [24] further examined the cyclic shear behaviour of cemented paste backfill using constant-volume direct simple shear tests. Their results indicated that curing age, cement content, and initial void ratio significantly affect the cyclic resistance and liquefaction potential of cemented paste backfill. Tan et al. [25] explored the mechanical behaviors of backfill under repetitive impact stressing. Under repeated cyclic impacts, backfill specimens generate prominent low-stress peaks before and after dynamic peak stress, which promotes the sufficient densification and sealing of inner tiny cracks and pores. Accordingly, transient mechanical strength and bearing capacity of backfills are improved to roughly two times the level under individual impact conditions, and the failure mode is dominated by compression cracking with a low fragmentation degree. Suazo et al. [26] used a split Hopkinson pressure bar to investigate the propagation and transmission of compressional stress waves in cemented paste backfill. In addition, existing studies have focused on triaxial creep [27,28], normal disturbance [29], low-frequency dynamic disturbance [30], and coupled dynamic-static loading [31].

However, most of the above studies have focused on single disturbance impacts or constant-amplitude cyclic loading, which fails to adequately simulate the complex engineering scenarios involving the coupling effect of two key variables: disturbance level (intensity) and disturbance frequency. In this study, quasi-static disturbance tests with various combination conditions of disturbance levels and frequencies were conducted. Combined with ultrasonic velocity monitoring and digital image correlation (DIC) techniques [32,33], the macroscopic mechanical behavior and full-field deformation characteristics of cemented tailings backfill under repeated stress disturbances were analyzed. With the adoption of scanning electron microscopy (SEM) and energy-dispersive spectroscopy (EDS) [34], the influence mechanism of disturbance history on the microstructure morphology, crack network, and chemical composition of hydration products of backfill was explored. Based on energy evolution analysis, a dissipated energy-centered damage variable and functional model was established, which effectively elucidates the damage accumulation mechanism of backfill under different disturbance histories from the energy perspective and provides a theoretical foundation for engineering practice.

## 2. Materials and Methods

### 2.1. Experimental Materials

**Figure 1:**
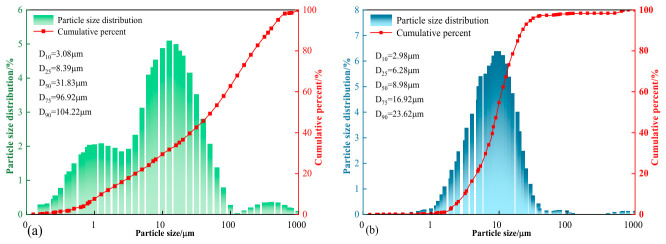
Particle size analysis: (**a**) tailings; (**b**) OPC42.5.

**Figure 2:**
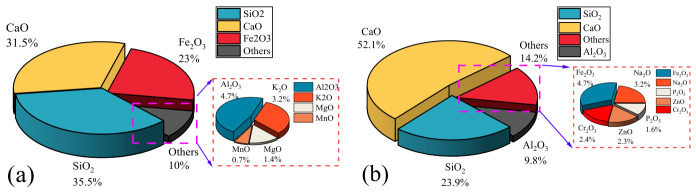
Chemical composition: (**a**) tailings; (**b**) OPC42.5.

**Figure 3:**
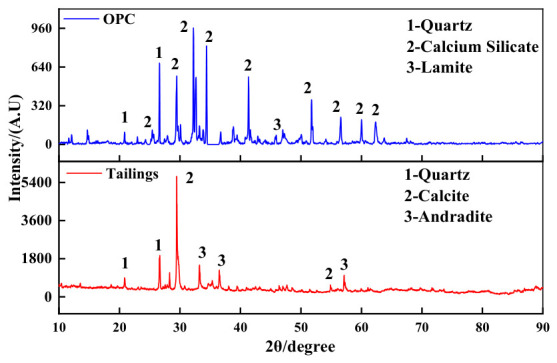
XRD patterns of materials.

This study mainly focuses on the effects of high- and low-frequency combined disturbances on the stability of backfill inside goafs. The raw materials of backfill consist of tailings collected from a lead-zinc mine in Chifeng, Inner Mongolia, China, commercially available ordinary 42.5 Portland cement, Zhucheng cement plant, Shandong Province and laboratory tap water. The tailings were oven (DHG-9070A, Shanghai, China)-dried and sieved through a 300-mesh sieve to remove coarse particulate impurities. The particle size tests of tailings and cement samples were carried out with an LS-POP laser diffraction particle tester, and relevant data are presented in Figure 1. The median particle diameter (D50) of the tailings reaches 31.83 μm, alongside a surface specific area of 715.3 m^2^/kg. The average particle size (D50) of the cement is 8.98 μm, with a specific surface area of 955.9 m^2^/kg. XRD (Dandong, China) and XRF (Kunshan, China) tests were adopted to characterize the phase composition and elemental constituents of the materials, and the relevant results are presented in Figure 2 and Figure 3. The mine tailings are dominated by SiO_2_ (35.5%), CaO (31.5%) and Fe_2_O_3_ (23%), mainly corresponding to quartz, calcite and other minerals. The cement primarily consists of SiO_2_ (35.5%), CaO (31.5%) and Al_2_O_3_ (9.8%), containing typical chemical components including C_3_S, C_2_S and CaO.

### 2.2. Sample Preparation

To reproduce real industrial application conditions, the multiple stress disturbances designed in this study simulate the dynamic mechanical environment encountered in deep multi-step mining. In actual backfill mining, the cemented tailings backfill (CTB) is repeatedly subjected to intermittent stress wave disturbances caused by frequent blasting and mechanical excavation in adjacent stopes during its curing period. The different stress disturbance levels (20–80% of static strength) represent the varying distances of the backfill from the blasting source, while the disturbance frequencies (1 to 4 times) correspond to the number of adjacent mining steps or discrete blasting events occurring during the early curing stages.

**Figure 4:**
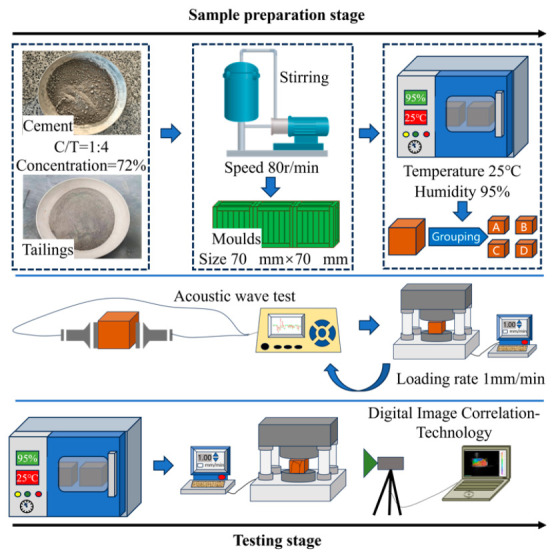
Experimental Flow Diagram.

To analyze the effect of combined multiple stress disturbances on the performance of the backfill, a backfill with a CT ratio of 1:4 and a mass fraction of 72% was prepared. Using the 3 d compressive strength σ_P_ (2.93 MPa, rounded to 3 MPa) of the preliminary test backfill as a reference, 17 sets of specimens were designed using different stress disturbance levels (20% σ_P_, 40% σ_P_, 60% σ_P_, 80% σ_P_) and disturbance frequency (1, 2, 3, 4) as variables. A total of 17 test groups were designed, with three specimens prepared for each group to minimize random errors. The specific mix ratios and test procedures are shown in Table 1. The materials were placed in a mixer in the specified proportions, mixed uniformly, and then poured into 70 × 70 × 70 mm cubic molds. The molds were placed in a constant temperature and humidity curing chamber (temperature 25 °C, humidity 95%). After 1 d, the specimens were demolded and continued to cure. At the curing stages of 3 d, 7 d, 14 d, and 21 d, the specimens were removed for multiple stress disturbances tests, respectively, and compressive strength testing was conducted at 28 d. The specific test procedure is shown in Figure 4.

### 2.3. Experimental Methods

(1) Non-metallic ultrasonic test: The equipment used is an NM-4B ultrasonic wave velocity analyzer operating with a transducer frequency of 50 kHz and an emission voltage of 500 V. Wave velocity tests were conducted on the specimens 3 d after initial curing and after each composite stress disturbance. Vaseline was applied evenly to both sides of the filling body to ensure proper contact with the sensor probe, and the data was recorded once the waveform stabilized. The average of the two test results was taken as the specimen’s test value [35].
(2) Uniaxial compressive strength (UCS) test: The multiple stress disturbance loading during the curing cycle and the definitive Uniaxial Compressive Strength (UCS) test. For the stress disturbance protocol applied at specific early curing ages (3 d, 7 d, 14 d, and 21 d), the specimen is placed in a 300 kN capacity electro-hydraulic servo universal testing machine. A displacement-controlled loading rate is set at 1 mm/min. The specimen is loaded to a predetermined pre-stress level and subsequently unloaded. The “disturbance frequency” is precisely defined as the number of these continuous axial load-unload cycles applied. Following the complete 28-day curing period, the definitive UCS test is conducted by continuously loading the specimens at 1 mm/min until macroscopic failure occurs, which provides the ultimate peak strength.
(3) Digital image correlation (DIC) test: White paint is sprayed evenly onto the side of the specimen, and black paint is used to apply evenly distributed black spots to the side of the CTB specimen. During loading, a high-frequency camera (operating at a capture rate of 15 frames per second with a spatial resolution of 2448 × 2048 pixels under constant LED illumination) records the displacement and motion of the black spots in real time. After processing withVIC–2D 7.2, the specimen’s failure and deformation characteristics are visualized through pixel-level changes in the displacement of the black spots [36].
(4) Scanning electron microscope (SEM) test: Select typical fragments from the fracture surface of the specimen and halt hydration by soaking them in anhydrous ethanol. Prepare the fragments into 5 mm × 5 mm pieces, wrap them in copper foil, carbon-coat them, and then examine them under a scanning electron microscope operating at an accelerating voltage of 20.00 kV and a working distance of 10 mm.

## 3. Results

### 3.1. Mechanical Properties of Backfill Under Multiple Stress Disturbances

#### 3.1.1. Stress-Strain Curve

**Figure 5:**
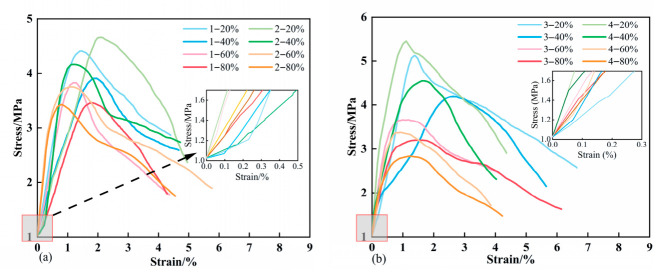
Stress-strain curves of the backfill under different disturbance times: (**a**) 1~2 times; (**b**) 3~4 times.

Figure 5 shows the full stress-strain curves of the specimens under different multiple stress disturbances. The stress-strain curves can be divided into four main stages: the microcrack compression stage, the elastic stage, the crack propagation stage, and the failure stage. When stress disturbances are applied during the curing period, lower-level disturbances cause the existing microcracks within the specimen to close. This occurs because the disturbance stress acts on the crack surfaces, causing them to move closer together and consolidate. As the frequency of disturbances increases, this compaction effect accumulates, gradually filling the microscopic voids and improving the overall density of the specimen, thereby acting as a compaction process. Consequently, during subsequent loading, the microcrack compression stage, which would normally require energy to close the cracks, becomes less pronounced, and the specimen rapidly enters the elastic stage.

When the applied stress is too high, not only does the high stress fail to effectively consolidate the microcracks, but it also causes significant stress concentration at the crack tips, driving the propagation of existing microcracks and the initiation of new cracks. This disrupts the internal structure of the specimen, leading to an increase in defects and consequently weakening its mechanical properties.

#### 3.1.2. Peak Intensity

**Figure 6:**
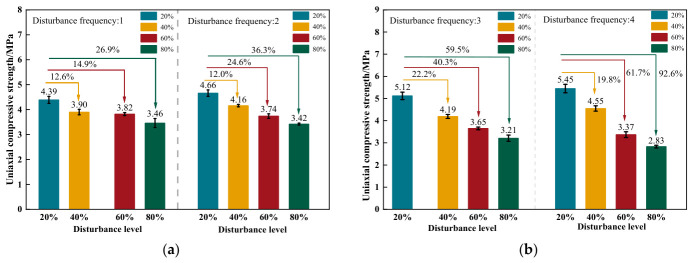
Uniaxial compressive strength under different stress disturbances: (**a**) 1~2 times; (**b**) 3~4 times.

Figure 6 illustrates the variation of ultimate strength for the backfill subjected to different multiple stress disturbances. When the disturbance level is 20% σ_P_, sample strength rises as the frequency of prestressing; when the disturbance level is 40% σ_P_, the rate of increase in backfill strength decreases significantly as the disturbance frequency increases. As the disturbance level increases further, the rise in disturbance frequency weakens the load-bearing capacity of the backfill; this weakening becomes more pronounced as the prestressing increases. For a single disturbance, compared to a disturbance level of 20%σ_P_, the strength of the backfill decreased by 12.6%, 14.9%, and 26.9% at different disturbance levels, respectively. At a disturbance level of 80%σ_P_, the strength of the backfill decreased from 3.46 MPa to 2.83 MPa as the number of disturbances increased; the strength was lowest when the disturbance frequency was 4 times. In contrast, after 4 disturbances at a disturbance level of 20% σ_P_, the backfill strength was 5.45 MPa, which was the highest strength among the specimens.

#### 3.1.3. Peak Strain and Modulus of Elasticity

**Figure 7:**
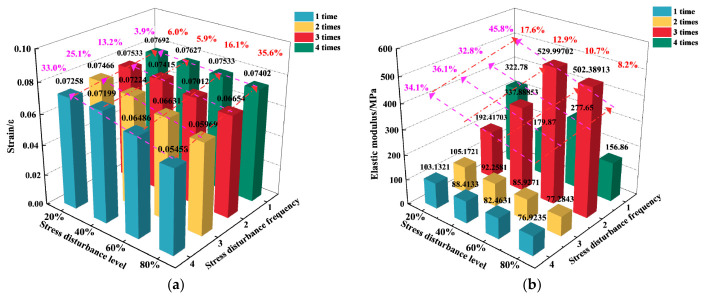
Peak strain and elastic modulus change trends: (**a**) peak strain; (**b**) elastic modulus change trends.

Figure 7 shows the variation in peak strain and elastic modulus under different multiple stress disturbances. In Figure 7a, the disturbance level exerts remarkable influence on the peak strain of cemented backfill, whereby the two exhibit an inverse relationship. As the prestressing load rises, the peak strain of cemented backfill slowly declines. When the disturbance frequency is 4 at 80%σ_P_, the peak strain decreases by 35.6% as the prestressing disturbance frequency of the grouted fill body increases from 1 to 4.

In Figure 7b, at the same disturbance frequency, the elastic modulus of backfill falls along with growing disturbance intensity, and a negative correlation is observed between the two. For the 4–20% specimen, the initial elastic modulus reached 103.13 MPa. Once the disturbance level increased to 40%σ_P_, 60%σ_P_, and 80%σ_P_, the elastic modulus decreased to 88.4133 MPa, 82.4631 MPa, and 76.9235 MPa, respectively, representing an overall decrease of 34.1%. The disturbance level affects the rigid performance of the cemented backfill, reducing its resistance to deformation. Under the same disturbance level, the elastic parameter of backfill shows negative correlation with the disturbance frequency. Greater disturbance frequency corresponds to smaller elastic modulus of samples. For the 1–80% specimen, the elastic modulus was 83.2152 MPa. When the disturbance frequency increased to 2, 3, and 4, the elastic modulus decreased to 79.2124 MPa, 77.2843 MPa, and 76.9235 MPa, respectively, representing an overall decrease of 8.2%. For specimens at 1–20% σ_P_, the elastic modulus decreased by 17.6% as the disturbance frequency increased from 1 to 4. The effect of disturbance frequency on the elastic modulus of the fill material is far less significant than the effect of disturbance level. During the actual curing period, attention should be paid to controlling the disturbance level to improve the deformation resistance of the specimens.

### 3.2. Analysis of Internal Damage Characteristics in Filled Bodies Under Different Multiple Stress Disturbances

#### 3.2.1. Wave Speed Characteristics Under Different Multiple Stress Disturbances

**Figure 8:**
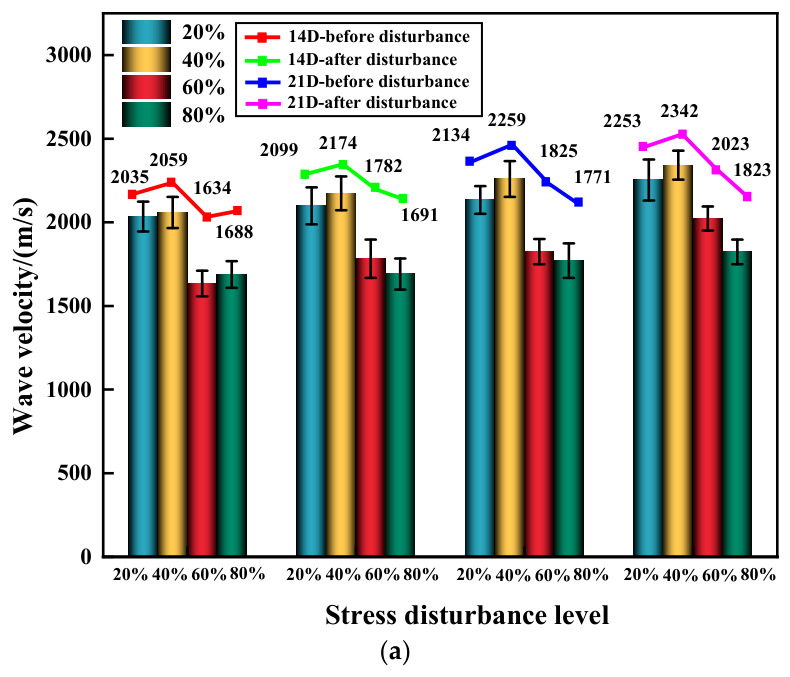

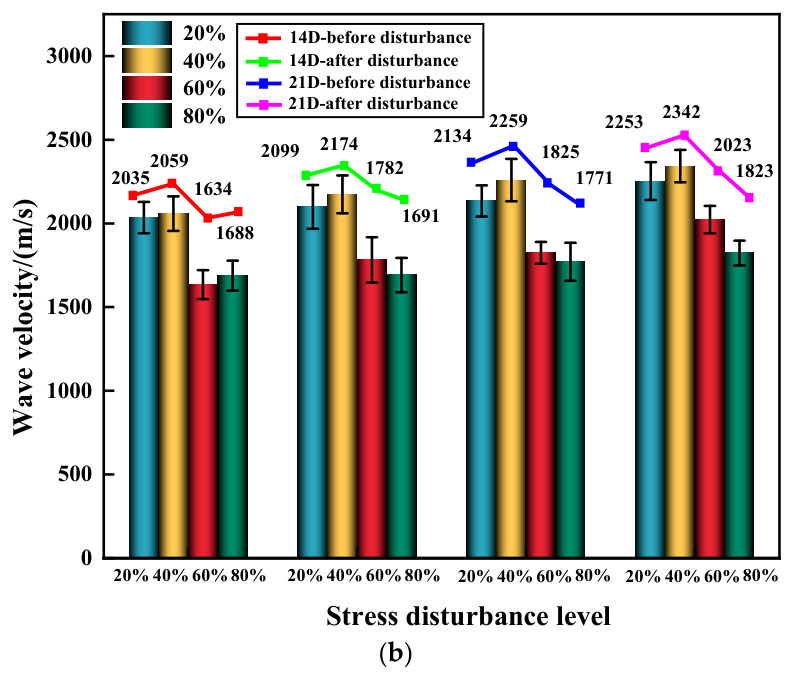
Wave speed test results: (**a**) Wave velocity after pre-stress at 3 d and 7 d; (**b**) Wave velocity after pre-stress at 14 d and 21 d.

Changes in ultrasonic wave velocity can reflect changes in internal cracks within the material in real time. As shown in Figure 8, wave velocity measurements indicate that the development of internal cracks in specimens during the early curing stage is related to the level of disturbance and the hydration reaction. This effect is more pronounced during the early stages of curing. Before and after the first disturbance at 3 d of curing, the wave velocity increased by 15.0%, 14.4%, 8.0%, and 8.4%, respectively, across the four disturbance levels. At 7 d of curing, internal hydration products further densified the pore structure of the matrix, resulting in an overall increase in wave velocity. However, at the 20% σ_P_ and 40% σ_P_ disturbance levels, the increase in wave velocity was higher than at the 60% and 80% levels. At 3 d of curing, after the specimen was subjected to prestressing, some water was exuded. The high stress inhibited subsequent hydration reactions within the specimen, resulting in a lack of sufficient cementitious products to support the particles, thereby reducing the wave velocity. The effect of hydration on wave velocity growth weakened at 14 d and 21 d of curing, but subsequent stress disturbances still disrupted the internal hydration products that had not yet matured.

#### 3.2.2. Energy Dissipation Analysis

The laws of thermodynamics indicate that energy conversion is an intrinsic aspect of changes in the physical properties of matter; therefore, the deformation and failure of a filled body under uniaxial loading must be accompanied by the accumulation and dissipation of energy. Assuming that no heat exchange occurs in the filled body during this process, according to the first law of thermodynamics:

(1) $$\Delta U=Q+W=\int_{0}^{\varepsilon }\sigma d\varepsilon$$

In the Equation, Δ*U* represents the work done by the load; *Q* represents the elastic energy accumulated by the CTB during the process from loading to failure, in kJ/m^3^; and *W* represents the energy dissipated by the CTB due to internal crack propagation and plastic deformation during this process, in kJ/m^3^. *σ* and *ε* represent the stress and strain experienced by the backfill, respectively.

(2) $$Q=\frac{\sigma^{2}}{2E}$$

**Figure 9:**
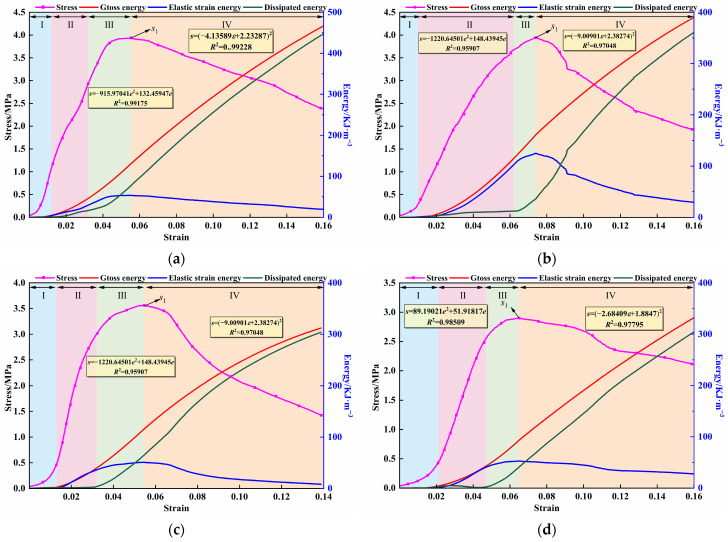
Energy evolution curve: (**a**) 1–60%; (**b**) 1–80%; (**c**) 4–60%; (**d**) 4–80%.

As shown in Equation (2), this is the formula for elastic energy, where *E* is the elastic modulus. By applying the above formula, it is possible to determine the variations in total energy, elastic strain energy, and dissipated energy of the specimen under uniaxial compression at different levels of disturbance and frequencies, as shown in Figure 9.

The trends in energy evolution of the specimens are essentially the same under different levels and frequencies of disturbance. In Stage I, intrinsic pores and cracks inside the specimen undergo compaction; during this stage, both the elastic strain energy and the dissipated energy are relatively low, with no clear growth pattern, and the energy conversion efficiency is low. In Stage II, elastic strain energy accumulates rapidly within the specimen. During this process, elastic strain energy is significantly higher than dissipated energy; the slight increase in dissipated energy is attributed to the closure of a small number of internal voids. In Stage III, the elastic strain energy within the specimen reaches its maximum value, and dissipated energy accumulates rapidly. When the prestress level is 60%σ_P_, the dissipated energy surpasses the elastic strain energy ahead of schedule during the yield stage. Subsequently, the process enters Stage IV (the post-peak failure or residual stage). During this final stage, macroscopic cracks coalesce to form penetrating failure bands. The load-bearing capacity of the specimen drops sharply, accompanied by an abrupt release of the stored elastic strain energy. Concurrently, the dissipated energy experiences a dramatic surge due to extensive internal structural friction and the sliding of macroscopic fracture surfaces, ultimately leading to the complete instability and structural failure of the backfill.

#### 3.2.3. Analysis of Damage Characteristics Based on Energy Dissipation Analysis

Damage to fillers is primarily caused by the accumulation of dissipated energy. When defining filler damage in terms of dissipated energy, the extent of damage can be characterized by the ratio of the internal dissipated energy of the specimen under uniaxial loading to the cumulative dissipated energy at the point of ultimate failure.

(3) $$D=\frac{W\left(\varepsilon \right)}{W_{max}\left(\varepsilon \right)}$$

In the equation, *D* represents the damage degree, *W*(*ε*) is the dissipated energy at a given point on the stress-strain curve, and *∑W*(*ε*) is the cumulative dissipated energy at the point of ultimate failure of the specimen. In Equation (3), *D* = 1 when the infill material is completely destroyed; however, in actual practice, the sample maintains a certain bearing strength even upon the point of ultimate failure, and internal elastic strain energy keeps transforming toward dissipated energy throughout the post-peak failure stage. Therefore, the damage degree is characterized based on the conversion rate of elastic strain energy to dissipated energy. In the actual calculation process, an appropriate reduction factor k must be selected to determine the damage level of the infill material that aligns with reality.

(4) $$k=\frac{Q_{max}-Q_{s}}{Q_{max}}=1-{\left(\frac{\sigma_{s}}{\sigma_{max}}\right)}^{2}$$

In this equation, *Q_max_* represents the peak elastic strain energy of the specimen under load, and *Q_s_* represents the elastic strain energy of the specimen that stabilizes during the post-peak failure stage under load. Combining Equations (3) and (4) yields:

(5) $$D=k\frac{W\left(\varepsilon \right)}{W_{max}\left(\varepsilon \right)}$$

**Figure 10:**
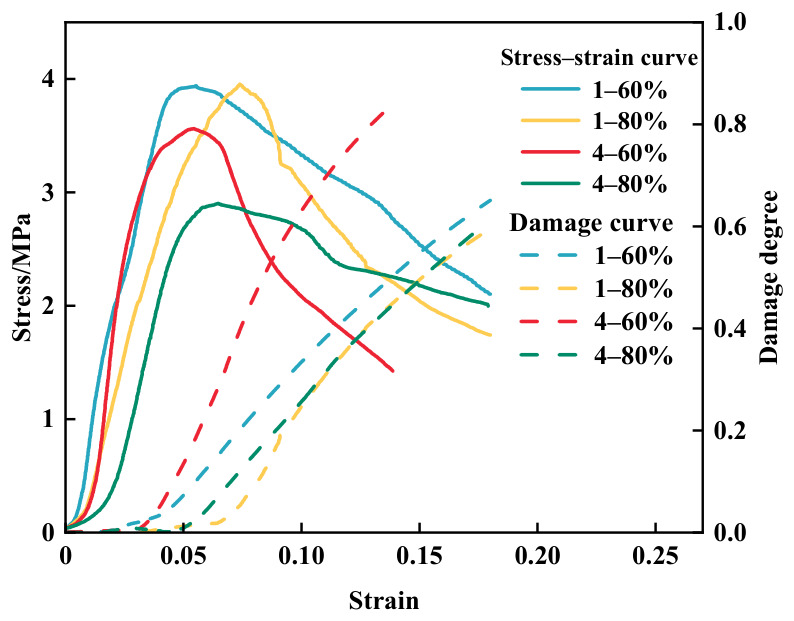
Sample damage curve.

The damage curve of the filled specimen calculated using the above formula is shown in Figure 10. Damage in the specimen primarily begins during the plastic stage and accelerates sharply after the stress peak. This is a typical damage characteristic of brittle-plastic materials, where plastic deformation initiates internal microdefects, and these microdefects propagate through the material after the peak, leading to macroscopic failure. Stress perturbation is the key driving factor in damage evolution; its magnitude and frequency jointly determine the initiation time, evolution rate, and ultimate extent of damage. Based on the stress-strain curve characteristics shown in Figure 5, it can be observed that when the stress level reaches 60% σ_P_, the specimens generally move beyond the elastic deformation stage, and internal microcracks enter a phase of accelerated propagation or even local yielding. Therefore, this study explicitly defines a disturbance level of 60% σ_P_ as a high-disturbance category. At this level, specimens in the 1–60% and 4–60% ranges begin to experience significant plastic damage as early as 0.02 strain. Additionally, compared to low-frequency disturbances, high-frequency disturbances result in greater damage at lower strains, with the 4–60% range exhibiting the highest relative damage severity. The internal damage caused by early disturbances provides stress concentrations and focal points during subsequent loading, accelerating damage evolution. The higher the initial damage level, the earlier damage initiates and the faster it develops under subsequent disturbance frequencies. At the same time, lower-level, high-frequency disturbances result in a higher degree of damage during subsequent failure. Each cycle introduces energy into the inner microstructure of the material; part of this energy is dissipated and accumulated in the form of plastic work and surface energy (crack propagation), allowing microdefects more time (or cycles) to nucleate, propagate, and connect. Consequently, when the same macroscopic strain is reached, the internal damage level is significantly higher than that of specimens subjected to low-frequency disturbances.

#### 3.2.4. Damage Constitutive Model Verification

According to the strain equivalence hypothesis, the damage constitutive model of a filling material under uniaxial loading can be expressed by:

(6) $$\sigma =E\varepsilon \left(1-D\right)$$

Prior to mechanical testing, the interior of the backfill contains numerous internal defects, such as microscopic cracks and voids. These defects are compacted during Stage I. To improve the accuracy of the damage constitutive model, the model is often considered in stages. Specifically, analysis of the stress-strain curve reveals that, prior to the elastic deformation stage, the curve increases logarithmically with increasing strain. By introducing the disturbance level influence coefficient m and the disturbance frequency influence coefficient *n* into Equation (6), Equation (7) is obtained.

(7) $$\sigma =\left\{\begin{array}{cc} \log_{m}\left[\frac{\left(m-1\right)\varepsilon }{\varepsilon_{c}}+1\right]\cdot E\varepsilon \left(1-k\frac{W\left(\varepsilon \right)}{W_{max}\left(\varepsilon \right)}\right) & 0<\varepsilon <\varepsilon_{c}\\ E\varepsilon \left(1-k\frac{W\left(\varepsilon \right)}{W_{max}\left(\varepsilon \right)}\right) & \varepsilon >\varepsilon_{c} \end{array}\right.\sigma =\left\{\begin{array}{cc} \log_{n}\left[\frac{\left(n-1\right)\varepsilon }{\varepsilon_{c}}+1\right]\cdot E\varepsilon \left(1-k\frac{W\left(\varepsilon \right)}{W_{max}\left(\varepsilon \right)}\right) & 0<\varepsilon <\varepsilon_{c}\\ E\varepsilon \left(1-k\frac{W\left(\varepsilon \right)}{W_{max}\left(\varepsilon \right)}\right) & \varepsilon >\varepsilon_{c} \end{array}\right.$$

**Figure 11:**
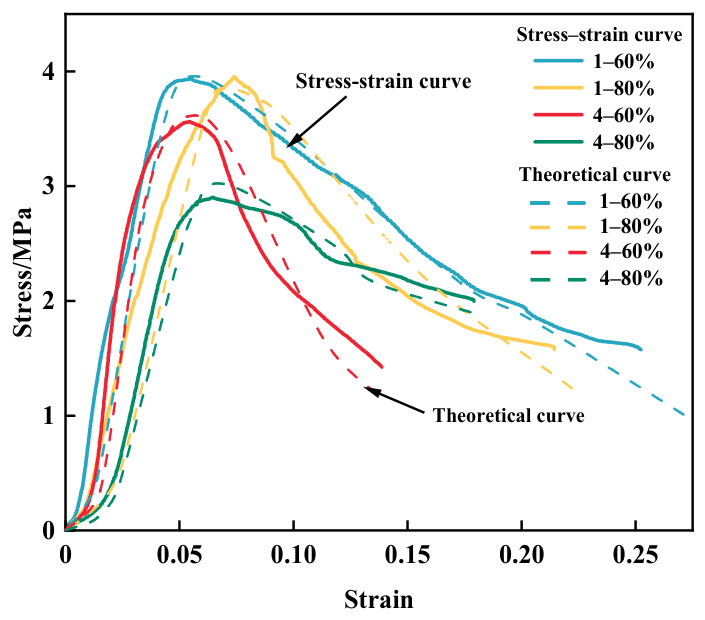
Damage constitutive model.

In the equation, *m* and *n* are constants obtained by fitting experimental data from Stage I specimens, and *ε_c_* is the strain at the end of Stage I. Damage-related evolution constitutive relation plotted derived from the equation appears shown in Figure 11. A comparison between the damage variation constitutive curve and the measured stress-strain profile indicates their changing patterns possess high similarity. Although there is a slight deviation in the post-peak stage, the general evolution paths are consistent. The theoretical damage evolution curve effectively describes the stress-strain changing law of the CTB, which proves the reliability of the piecewise damage evolution constitutive relation established herein has been verified. Compared to traditional constitutive models based on empirical fitting or single-strain equivalence, the damage calculation framework built in this study based on energy dissipation can more profoundly reflect the physical and thermodynamic essence of microdefects within the filler material, from initiation and propagation to through-failure. Since energy dissipation is a direct measure of irreversible material aging, this model exhibits better robustness for different stress paths and has broad applicability.

### 3.3. Failure Mode and Fracture Morphology Analysis

#### 3.3.1. DIC Test

The videos recorded during uniaxial compression tests were processed and converted into sequential images with an interval of 500 milliseconds per frame, which were then imported into VIC-2D 7.2 software for DIC analysis.

**Figure 12:**
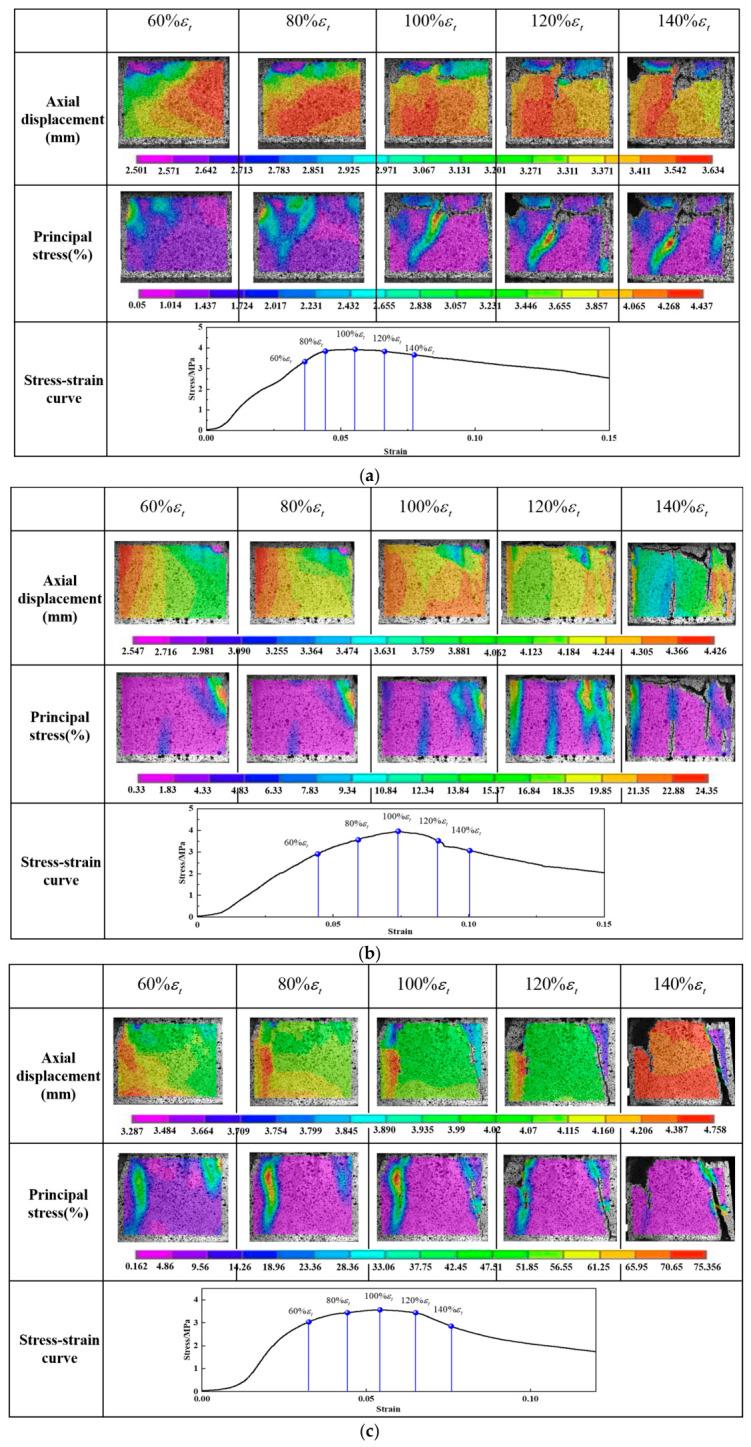

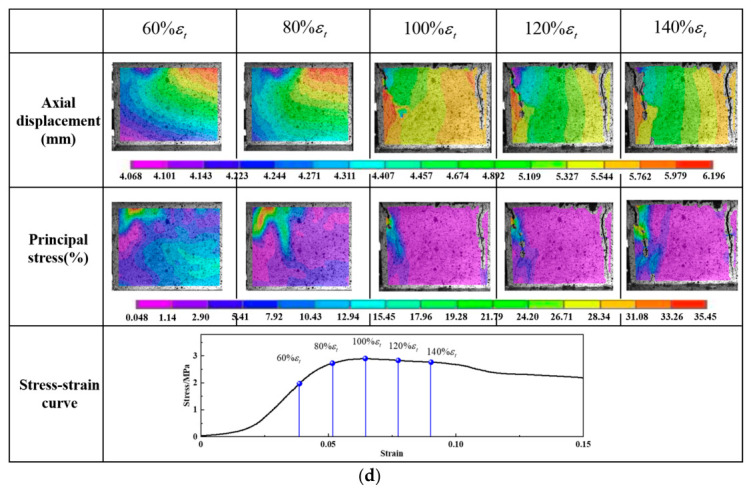
Axial strain contour map and evolution of principal stress for specimens in under uniaxial compression testing: (**a**) 1–60%; (**b**) 1–80%; (**c**) 4–60%; (**d**) 4–80%.

Figure 12a–d presents the axial strain contours and the evolution of the first principal stress of CTB specimens under uniaxial compression. In the axial strain nephograms, the purple regions correspond to areas undergoing axial compressive deformation, while the red regions represent zones with lateral deformation. For the first principal stress nephograms, purple indicates low-stress areas and red denotes high-stress areas. $\varepsilon_{t}$ is defined as the strain at the peak stress. The range from 60% to 160% $\varepsilon_{t}$ was selected to analyze the failure characteristics of the specimens.

The DIC results further reveal the progressive localization and instability mechanism of CTB under different disturbance histories. As shown in Figure 12a–d, the specimens generally experienced a failure process from local strain concentration to crack initiation, propagation, coalescence, and final instability. Under low-frequency disturbance, the strain localization zone developed relatively gradually, and the failure path was mainly controlled by the progressive extension of localized shear cracks. With increasing disturbance level, axial strain concentration became more pronounced, and the principal-stress concentration zone evolved more rapidly into a penetrating failure band, indicating that high-level disturbance promoted crack initiation and accelerated structural deterioration.

Under high-frequency disturbance, repeated loading further intensified the cumulative damage effect. The strain and principal-stress fields became more heterogeneous, and multiple local deformation zones tended to connect during loading. In particular, the specimen subjected to high disturbance level and high frequency exhibited the most obvious non-uniform deformation and rapid post-peak instability. Therefore, the DIC observations confirm that disturbance level governs the degree of damage localization, whereas disturbance frequency accelerates the accumulation and coalescence of microcracks. This conclusion is consistent with the stress–strain response, energy dissipation behavior, and damage evolution analysis, indicating that high-level and high-frequency composite disturbances significantly weaken the deformation resistance and structural integrity of CTB.

#### 3.3.2. Microstructural Analysis

**Figure 13:**
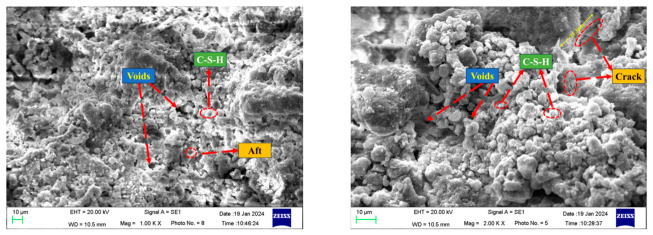
SEM pictures of fracture surface.

Figure 13 shows SEM images of the fracture surfaces of specimen 1–80% content after failure. The fracture surfaces reveal plentiful hydrated substances, including C-S-H gel and ettringite (AFt phase). These hydration products are distributed within the pores and around cracks on the fracture surfaces; some even directly fill the internal pores and cracks. These hydration products possess excellent interfacial bonding properties, holding the internal structure of the CTB together. Their widespread distribution significantly en-hances the strength of the CTB. Dynamic loading environments and internal stress concentrations significantly alter the local chemical coordination, hydration dynamics, and nanoscale precipitation of calcium silicate hydrates [37], repeated stress applications accelerate the coalescence of microcracks and facilitate internal chemical migration within the matrix, which directly modifies the nanomechanical integrity and bulk macroscopic performance of the material [38]. Microcracks with a maximum length of 21.3 μm were observed on the fracture surfaces of the 1–80% specimen; however, there were few sur-rounding hydration products. It can be preliminarily concluded that these cracks were generated during subsequent uniaxial loading and are not internal defects originating from the early prestressing disturbance stage.

**Figure 14:**
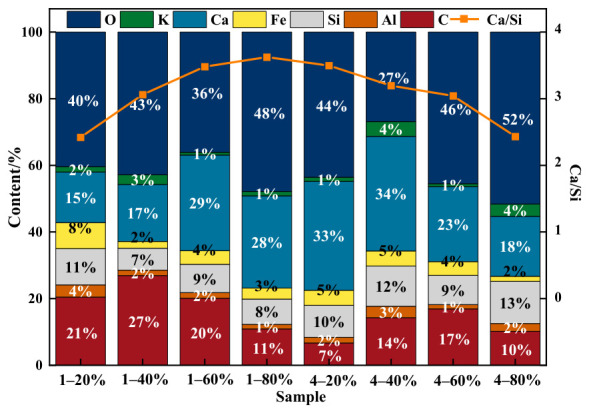
Analysis of element proportions in different samples.

According to the EDS analysis shown in Figure 14, the major elements in the fracture surface of the CTB are C, O, Ca, Si, Fe, Al, and K. The O content is relatively high, and the C element primarily originates from the carbon sputtering process during the SEM sample pretreatment. The results of the elemental analysis show that when the disturbance frequency is 1 and the disturbance level is 20% σ_P_, the Ca/Si ratio is 1.39, and the calcium-to-silicon ratio increases as the disturbance level increases. This indicates that prestressing loads have influenced the early hydration reactions of CTB. This influence may have promoted the formation of Ca(OH)_2_ and ettringite during hydration, leading to an increase in the Ca/Si ratio observed in the surface scanning elemental analysis.

## 4. Conclusions

This research explores mechanical performance and failure features of CTB under multiple stress disturbances, as well as influences of prestressing perturbation magnitudes alongside vibration frequency on bearing capacity, strain features, and energy evolution mechanisms of CTB. Unconfined uniaxial compression tests, NM-4B non-metallic ultrasonic velocity tests, DIC, and SEM tests were conducted, leading to the following conclusions:

(1) Under early, minor disturbances, increasing the disturbance frequency can enhance the peak strength of the backfill. However, higher disturbance levels and higher disturbance frequencies degrade the backfill, resulting in reduced peak strain and elastic modulus; among these factors, the disturbance intensity exerts a stronger influence on the degradation of deformation capacity.
(2) Stress disturbances can compact the internal structure of the backfill, reduce porosity, and improve early-stage density; at the same time, the early-stage hydration reaction itself also helps reduce porosity. However, excessive disturbance can cause free water to leach out, disrupting the hydration process and thereby undermining its ability to improve structural density.
(3) During loading, the backfill material undergoes three stages: consolidation, elastic accumulation, and plastic failure. At high disturbance levels (≥60%), the yield stage is prolonged, dissipated energy exceeds elastic energy earlier, and damage initiation occurs sooner while its progression accelerates. A damage function based on dissipated energy and modified by elastic strain energy can more accurately characterize the actual damage process. Validation of the piecewise damage constitutive model indicates that it effectively reflects the stress-strain response, reveals the coupled effects of disturbance level and frequency on damage evolution, and provides a theoretical basis for the dynamic damage assessment of fillers.
(4) Under low-frequency disturbance, an increase in the disturbance level leads to the enhanced formation of Portlandite (Ca(OH)_2_) and Ettringite, resulting in a higher Ca/Si ratio. Conversely, under high-frequency disturbance, an increase in the disturbance level significantly promotes the formation of C-S-H gel, resulting in a lower Ca/Si ratio.
(5) This study clarifies the mechanisms by which disturbance levels and frequencies exert their effects. It provides direct guidance for the design of backfill material mix proportions, early-stage curing control, and dynamic assessments of mining area stability in highly disturbed regions and offers a theoretical basis for risk suppression and governance of rock dynamic hazards within deep underground mines alongside the resource utilization of solid waste. Future work should involve multi-scale observations, long-term multi-field coupled experiments, and simulations of complex disturbance pathways, while integrating damage models into numerical simulations to enhance predictive capabilities; concurrently, research and development of disturbance-resistant reinforced materials should be pursued.

## Figures and Tables

**Table 1:** Experiment design.

Name	Disruption Frequency	Disruption Grade	CT Ratio	Mass Fraction (%)	28 d Strength (MPa)
Control	0		1:4	72%	4.47
1–20%	1	20% σ_P_	1:4	72%	4.58
1–40%		40% σ_P_			4.12
1–60%		60% σ_P_			4.09
1–80%		80% σ_P_			3.84
2–20%	2	20% σ_P_	1:4	72%	4.65
2–40%		40% σ_P_			4.33
2–60%		60% σ_P_			3.95
2–80%		80% σ_P_			3.58
3–20%	3	20% σ_P_	1:4	72%	5.02
3–40%		40% σ_P_			4.47
3–60%		60% σ_P_			3.85
3–80%		80% σ_P_			3.36
4–20%	3	20% σ_P_	1:4	72%	5.36
4–40%		40% σ_P_			4.59
4–60%		60% σ_P_			3.55
4–80%		80% σ_P_			2.98

## Data Availability

The original contributions presented in this study are included in the article. Further inquiries can be directed to the corresponding author.
